# Editorial for the Special Issue on Flexible Sensors and Actuators for Biomedicine

**DOI:** 10.3390/mi14122184

**Published:** 2023-11-30

**Authors:** Jingyan Zhang, Mengdi Han

**Affiliations:** Department of Biomedical Engineering, College of Future Technology, Peking University, Beijing 100871, China; 2301112473@pku.edu.cn

Flexible sensors and actuators typically rely on functional materials with low Young’s moduli or ultrathin geometries. These materials undergo mechanical or electric changes in reaction to external stimuli, such as pressure, temperature, and electromagnetic fields [1,2,3], enabling sensing or motion capabilities. These flexible devices find widespread applications in wearable devices, implantable medical devices, and micro-robotics for biomedical monitoring, diagnosis, and therapies. Flexible sensors continuously monitor human physiological indices such as blood pressure, heart rate, blood oxygen saturation level, neural electrical signals, and the chemical composition of body fluids [4,5,6,7]. Meanwhile, flexible actuators are commonly employed in drug delivery microsystems, surgical robotics, prosthetics, and other assistive devices [8,9,10]. This Special Issue comprises five contributions, including research and reviews on biochemical sensors, self-powered sensors driven by nanogenerators, and soft robotics for colonoscopes.

Biochemical sensors are devices that detect and measure the presence or concentration of specific chemical substances or biomolecules. These sensors have broad applications in various fields, including environmental monitoring, food safety, industrial production, and biomedicine [11,12,13,14]. For biomedical applications, biochemical sensing provides measurements of specific chemicals in the human body for health diagnosis [15]. For example, the continuous monitoring of glucose concentration in blood, interstitial fluid, or sweat can serve as an effective means for the early diagnosis and management of diabetes [16]. Biochemical sensors with high specificity typically incorporate biological molecules (enzymes [17], antibodies [18], and aptamers [19]) modified on the surface of the electrode. Such modifications enable specific binding with target molecules, thereby enabling highly selective chemical sensing and reducing interference from other substances. Aptamers, derived using an in vitro selection technique known as systematic evolution of ligands via exponential enrichment (SELEX), are structured oligonucleotide sequences with a specific recognition capability and high affinity for corresponding target molecules such as proteins, viruses, bacteria, cells, small molecule compounds, heavy metal ions, etc. In contrast to antibodies, aptamers offer advantages such as a shorter screening cycle, enhanced thermal and chemical stability, and the capability to bind to diverse targets [20]. Nguyen et al. [21] designed a simple, rapid, and ultrasensitive colorimetric aptasensor for detecting anatoxin-a (ATX-a). The specific binding of ATX-a to aptamers absorbed on the surface altered the aggregation state of gold nanoparticles, resulting in a color change in the solution. Using an ultraviolet/visible spectrophotometer, researchers measured changes in ATX-a concentration through absorbance variations, providing a rapid method for detecting water quality contaminated by microbial pollution. In a separate study, Chai et al. [22] demonstrated that P-doped carbon quantum dots (CQD) wrinkled and damaged bacteria through electrostatic interactions. The results suggested effective antibacterial activity of the P-doped CQD against *Escherichia coli* (*E. coli*) and *Staphylococcus aureus* (*S. aureus*). Furthermore, these nano-materials exhibited high biocompatibility and photostability, providing potential applications in bacterial infection treatment and bioluminescence sensors. In a review article, Tang et al. [23] summarized wearable sensors and systems for pH and temperature detection in the context of wound healing monitoring. Integrating pH and temperature sensors with flexible fabrics and actuators enabled real-time diagnostics and precise drug delivery. The wearable pH and temperature sensors demonstrated high sensitivity and reliability, with the potential for continuous monitoring during wound healing. In summary, biochemical sensors can rapidly and precisely detect complex solvents. Achieving high specificity and sensitivity to ensure accurate detection of target molecules is vitally important, especially for complex biological systems where many chemicals coexist.

Flexible sensors generally require batteries for measurement, signal processing, and data transmission. However, batteries usually have a larger, rigid form factor and limited operating time, which can hinder the portability, comfort, and long-term implantation of flexible sensors [24,25]. Self-powered flexible systems based on nanogenerators aim to overcome these challenges. These nanogenerators exploit the piezoelectric or triboelectric effect to convert mechanical energy into electricity, thus offering an alternative solution for power supply to micro-devices or sensors [26,27]. Yang et al. [28] provided a comprehensive overview of the evolution of piezoelectric/triboelectric nanogenerators, delving into materials and structural designs for both types of nanogenerators. The authors highlighted the biocompatibility and flexibility of the materials for better adherence to the skin surface and the organs inside the body. Regarding the device structure, the authors illustrated a diverse set of designs, such as three-dimensional structures, fabric structures, and thin-film structures, to enhance the output performance of nanogenerators. Finally, the authors summarized the broad applications of nanogenerators in wearable and implantable electronic devices, including motion detection, wound repairing, battery-less cardiac pacemakers, and in vivo health monitoring.

While flexible sensors have shown remarkable applications in biomedicine, they fall short in generating motion or offering mechanical stimulation for more interventional therapies. In such cases, soft actuators are often capable of producing motion [29] or deformation [30] under various stimuli such as mechanical input, laser irradiation, electric and magnetic fields, temperature variation, etc. [31,32,33]. These actuators are generally small, possess flexibility similar to biological tissues, and thus enable precise targeting and intervention in biomedical applications. Examples include micro-robots for precise drug delivery and balloon catheters for vascular occlusion therapy [34]. Chen et al. [35] proposed a robot colonoscope that resembles a caterpillar, capable of contracting, expanding, and turning in the horizontal, straight, or inclined porcine colons through anchoring and elongation units. The robot’s exterior, composed of soft rubber and balloons, prevents damage to the colon wall and alleviates discomfort. This robot can perform all functions of traditional colonoscope instruments, such as biopsies, inflation, and water jet surgery, presenting broad clinical application prospects. Although soft actuators have demonstrated various applications, the inherent properties of soft materials sometimes limit their performance [36]. Further research and improvements can be made in the output density, latency characteristics, and long-term stability.

This Special Issue explores flexible sensors and actuators for diverse biomedical applications. This editorial briefly introduces these works and overviews the working mechanisms, application scenarios, and potential challenges of representative soft flexible sensors and actuators. We thank all the contributors for submitting their papers to this Special Issue. We also thank all the reviewers for dedicating their time to help improve the quality of the submitted papers.
